# Can clinical guidelines reduce variation in transfusion practice? A pre–post study of blood transfusions during cardiac surgery

**DOI:** 10.1111/vox.13751

**Published:** 2024-10-14

**Authors:** Adam Irving, Anthony Harris, Dennis Petrie, Daniel Avdic, Julian Smith, Lavinia Tran, Christopher M. Reid, Zoe K. McQuilten

**Affiliations:** ^1^ Centre for Health Economics, Monash Business School Monash University Melbourne Victoria Australia; ^2^ Transfusion Research Unit, School of Public Health and Preventive Medicine Monash University Melbourne Victoria Australia; ^3^ Department of Economics Deakin University Melbourne Victoria Australia; ^4^ Department of Surgery School of Clinical Sciences at Monash Health, Monash University Melbourne Victoria Australia; ^5^ Department of Cardiothoracic Surgery Monash Health Melbourne Victoria Australia; ^6^ Centre of Cardiovascular Research and Education in Therapeutics School of Public Health and Preventive Medicine, Monash University Melbourne Victoria Australia; ^7^ School of Population Health Curtin University Perth Western Australia Australia; ^8^ Department of Haematology Monash Health Melbourne Victoria Australia

**Keywords:** cardiac surgery, clinical guidelines, patient blood management, variation in care

## Abstract

**Background and Objectives:**

Previously published studies have consistently identified significant variation in red blood cell (RBC) transfusions during cardiac surgery. Clinical guidelines can be effective at improving the average quality of care; however, their impact on variation in practice is rarely studied. Herein, we estimated how variation in RBC use across cardiac surgeons changed after the publication of national patient blood management guidelines.

**Materials and Methods:**

We performed a pre–post study estimating change in variation in RBC transfusions across 80 cardiac surgeons in 29 hospitals using a national cardiac surgery registry. Variation across surgeons was estimated using fixed‐effects regressions controlling for surgery and patient characteristics and an empirical Bayes shrinkage to adjust for sampling error. RBC use was measured by three metrics—the total number of units transfused, the proportion of patients transfused and the number of units transfused, conditional on receiving RBC.

**Results:**

The primary analysis utilized 35,761 elective cardiac surgeries performed between March 2009 and February 2015 and identified a 24.5% reduction (*p* < 0.0001) in mean total units transfused accompanied by a 37.2% reduction (*p* = 0.040) in the variation across surgeons. The reduction in mean total units was driven by both the proportion of patients transfused and the number of units transfused, conditional on receiving RBC, while the reduction in variation was only driven by the latter.

**Conclusion:**

In our study of RBC transfusions across cardiac surgeons, the surgeons who used more RBC in the pre‐guideline period experienced larger reductions in RBC use after the guidelines were published.

**Highlights**

- Clinical guidelines can be effective at improving the average quality of care; however, their impact on variation in care is rarely studied.
- We quantified how variation in transfusion practice changed after the publication of national patient blood management guidelines in 2012.
- We identified a reduction in variation across surgeons due to larger responses from surgeons who previously transfused more frequently.

## INTRODUCTION

Red blood cell (RBC) transfusions are commonly administered during cardiac surgery due to significant blood loss during and following the procedure; however, studies have consistently shown that there is significant variation in the use of RBCs, even after adjusting for patient factors that may contribute to the need for transfusion. For example, large variations in RBC transfusions have been identified across hospitals in the United States [1, 2, 3, 4], Australia [5] and elsewhere [6] and across surgeons in the United States [7] and Canada [8].

Variation in the healthcare delivered by providers is a widespread and persistent phenomenon, and a source for concern when deviations from what is currently considered to be ‘best practice’ are unrelated to patient need or preferences. Some of the variation at the patient‐level may be due to chance factors (e.g., rare, severe adverse events), but some may also represent true underlying variation in performance. This carries over to variation at the provider‐level which may be due to not only specific providers treating certain types of patients (e.g., a surgeon preferring to only operate on younger patients) but also variation in their performance. Wennberg et al. [9] define unwarranted variation to be variation that cannot be explained by patient heterogeneity, medical science or patient preferences. RBC transfusions during cardiac surgery are preference‐sensitive care—an intervention for which the risks, benefits and attitudes may differ across patients. Given that a central objective of healthcare systems is to provide appropriate care, reducing unwarranted variation across providers represents an opportunity to improve the overall quality of care and minimize unnecessary resource use [10].

By raising the awareness of the latest evidence, clinical guidelines aim to improve quality of care. While often assessed for their direct impact on changing average practice, the effectiveness of clinical guidelines as a strategy to reduce unwarranted variations across providers is yet to be established. As these two measures are statistically independent, it is not possible to infer a change in variation from a change in average practice. The objective of this study was to quantify how variation across surgeons in the use of RBC transfusions during cardiac surgery changed after publication of national patient blood management clinical guidelines.

The ideal approach to determining whether the clinical guidelines reduced variation in RBC transfusions would be a large randomized controlled trial, most likely clustered at the hospital level. However, such a trial is impractical, and instead clinical guidelines are generally developed and implemented at a national level. It is possible to use observation evidence in lieu of clinical trial data; however, there are methodological limitations that prevent strong causal conclusions from such analyses. Nonetheless, utilizing observational data we hypothesised that we would observe, on average, a larger response to the clinical guidelines in those surgeons who used more RBCs in the pre‐guideline period. Such a response would result in an average pattern of care that more closely conforms to the guidelines and a reduction in variation across surgeons. However, this pattern of response is not guaranteed if the clinical guidelines did not change the practice of high blood use surgeons.

## MATERIALS AND METHODS

### Study design

We performed a pre–post analysis using national cardiac surgery registry data from 29 hospitals in Australia. Ethics approval for the study was provided by Monash University (project ID 9097) in January 2021.

### Clinical guidelines

The patient blood management (PBM) clinical guidelines for perioperative care were published in March 2012 by Australia's National Blood Authority (NBA), who manage the national blood supply [11]. The NBA guidelines were made freely available online and printed copies were delivered free‐of‐charge to hospital departments who requested them. The NBA also developed a non‐mandatory eLearning module which was advertised through their own and hospital channels to all relevant hospital staff. A complete list of the guideline recommendations is included in Appendix S1.

There were no direct financial incentives offered to adhere to the guidelines. In most Australian states, blood products are paid for by the federal and state governments, not by the hospital. However, two states have devolved budgets for blood products, introducing an indirect financial incentive for hospitals to reduce blood use; however, previously published research did not find any difference in adherence to the guidelines in those states with indirect financial incentives and those without [12].

While there has been no formal assessment of hospital or surgeon fidelity to the guidelines by the NBA, a previously published interrupted time series analysis found that these guidelines were effective in reducing the average number of RBC, platelet and fresh‐frozen plasma transfusions during cardiac surgery admissions [12]. The analysis was able to detect a significant change in average practice at the time the guidelines were published but did not specifically evaluate changes in variation in care.

### Data source

This study used surgery‐level data routinely collected by the Australian & New Zealand Society of Cardiac & Thoracic Surgeons (ANZSCTS) Database Program. The ANZSCTS Database is a clinical quality registry designed to be able to monitor the delivery of cardiac surgery across Australia and New Zealand. Hospitals participating in the database submit pre‐defined data on all cardiac surgeries performed, including patient demographics, surgical history, pre‐ and intra‐operative medication, post‐operative outcome data and de‐identified surgeon codes. There are currently 26 public and 35 private hospitals contributing to the ANZSCTS Database including hospitals from all states and territories of Australia except the Northern Territory. As cardiac surgery is a complex procedure not commonly performed in regional hospitals the ANZSCTS Database is largely comprised of metropolitan hospitals. Data are manually entered into the ANZSCTS Database by hospital staff at the end of the procedure and at the end of 30‐day follow‐up using hospital medical records. The data are centrally checked by Monash University staff for errors, with requests for outliers to be reconfirmed by hospital staff.

### Outcome

We compared variation in RBC transfusions across cardiac surgeons using three metrics—the total number of RBC units transfused (including zeros), the proportion of patients who received a transfusion and the number of RBC units transfused, conditional on receiving blood (excluding zeros).

There were large clinical trials of liberal versus restrictive transfusion strategies in cardiac surgery published in the years following the publication of the clinical guidelines. The results of one of the largest trials, the transfusion indication threshold reduction (TITRe2) trial [13], were published in March 2015. To avoid contamination with the effects of the TITRe2 trial we compared variation in RBC transfusions across cardiac surgeons in the 3 years preceding the guidelines (March 2009–February 2012) was compared with the variation in the 3 years following the guidelines (March 2012–February 2015).

### Statistical analysis

Differences in surgery and patient characteristics were assessed to determine the similarity between the periods. Relying on the large sample size and the central limit theorem, we did not exclude outliers and tested means of all continuous data regardless of normality using the *t* test, medians using the Mann–Whitney *U* test and rates using Fisher's exact test. These characteristics formed the vector of control variables for all regressions and were the same as a previous predictive model of RBC transfusions in cardiac surgery [14].

Evaluating the change in the observed variance in RBC transfusions across cardiac surgeons before and after the guidelines is likely to be biased due to patient heterogeneity and sampling error. Patient heterogeneity, the fact that surgeons do not treat an identical set of patients, will produce differences in practice due to differences in observed or unobserved patient risk factors. In addition, sampling error will bias the observed variation upwards due to statistical uncertainty in the estimates of average RBC use across surgeons. We, therefore, estimated variation before and after the clinical guidelines were published using a novel method—a fixed‐effects regression model complemented by an empirical Bayes shrinkage [15]. The fixed‐effects regression includes patient risk factors to control for patient heterogeneity and surgeon fixed effects for the pre‐ and post‐guideline periods. The change in the estimated surgeon fixed effects will account for time‐invariant observed and unobserved surgeon heterogeneity, such as prevailing opinions regarding RBC transfusions. The subsequent empirical Bayes shrinkage adjusts the estimated surgeon fixed effects (i.e., their baseline blood use) for sampling error by ‘shrinking’ the posterior estimate towards the overall estimated mean across all surgeons. The degree of shrinkage will be largest for those surgeon effects that are more statistically uncertain [16]. In addition to empirical Bayes shrinkage, only data from surgeons who performed at least 10 surgeries in both the pre‐ and post‐guideline periods were included to improve the precision of estimates.

As many of the guideline's recommendations concern managing pre‐operative care, the sample for the primary analysis was restricted to elective cardiac surgeries only, defined in the ANZSCTS Database as procedures that could be deferred without risk of compromised cardiac outcome. The causal logic of the pre‐operative PBM recommendations is that by pre‐emptively optimizing blood volume, red cell mass and tolerance of anaemia, patients can withstand a greater degree of intra‐operative bleeding without the need for intra‐operative or post‐operative RBC transfusions. To quantify the robustness of the results to this restriction, we also performed a secondary analysis of non‐elective surgeries, excluding emergency and salvage operations. As the database contained very little missing data (<1.0% for all variables of interest), we performed a complete case analysis only. All analyses were conducted in Stata version 17 [17]. Kernel density plots were used to graphically present the distributions of RBC transfusions across surgeons. The Appendix S1 includes additional details regarding the fixed‐effects regressions, the empirical Bayes shrinkage and the interpretation of kernel density plots.

## RESULTS

For all analyses, the 6‐year sample was restricted to surgeons who performed at least 10 surgeries before and after the guidelines. For the primary, elective surgery analysis, this resulted in 80 surgeons who performed 35,761 surgeries, 17,049 in 26 hospitals and 18,712 in 29 hospitals in the pre‐ and post‐guideline periods, respectively. For the secondary, non‐elective surgery analysis, this resulted in 75 surgeons who performed 12,098 surgeries, 6361 and 5737 in the pre‐ and post‐guideline periods, respectively.

Table 1 presents the surgery and patient characteristics for the primary analysis. Patients were predominantly male (71.3%), with a mean age of 66.3 years. The majority (62.7%) underwent coronary artery bypass grafting, 48.1% underwent valve surgery, 14.6% underwent both and 20.1% had undergone previous cardiac surgery. Tests for differences in characteristics between the periods revealed some small but statistically significant differences. All patient characteristics in Table 1 were included as control variables in the regressions.

**TABLE 1:** Surgery and patient characteristics—elective surgeries.

Characteristic	Pre‐guideline	Post‐guideline	*p*‐Value
Surgeries	17,049	18,712	N/A
Surgeons	80	80	N/A
Hospitals	26	29	N/A
Coronary artery bypass	11,036 (64.8%)	11,396 (60.9%)	<0.0001
Valve surgery	7958 (46.7%)	9238 (49.4%)	<0.0001
Age, years, mean ± SD	66.1 ± 13.2	66.4 ± 13.0	0.018
Male, *n* (%)	12,089 (70.9%)	13,417 (71.7%)	0.097
BMI, kg/m^2^, mean ± SD	28.5 ± 5.5	28.6 ± 5.6	0.041
Cardiac catheterisation, *n* (%)	15,111 (89.1%)	16,996 (91.0%)	<0.0001
Cardiogenic shock, *n* (%)	90 (0.5%)	78 (0.4%)	0.124
Congestive heart failure, *n* (%)	3809 (22.4%)	2750 (20.0%)	<0.0001
Cerebrovascular disease, *n* (%)	1924 (11.3%)	1758 (9.4%)	<0.0001
Diabetes, *n* (%)	4829 (28.4%)	5228 (28.0%)	0.404
eGFR, mL/min/1.73 m^2^, mean ± SD	74.0 ± 33.1	77.2 ± 33.3	<0.0001
Infective endocarditis, *n* (%)	287 (1.7%)	311 (1.7%)	0.869
Myocardial infarction, *n* (%)	4912 (28.9%)	4952 (26.5%)	<0.0001
Peripheral vascular disease, *n* (%)	1587 (9.3%)	1501 (8.0%)	<0.0001
Respiratory disease, *n* (%)	2271 (13.4%)	2412 (12.9%)	0.224
Dialysis, *n* (%)	236 (1.4%)	211 (1.1%)	0.029
Intra‐aortic balloon pump, *n* (%)	373 (2.1%)	397 (2.1%)	0.638
Previous cardiac surgery, *n* (%)	3357 (19.7%)	3830 (20.5%)	0.068
Angina–CCS classification, *n* (%)			<0.0001
0	7111 (42.4%)	8649 (46.3%)	
1	1976 (11.7%)	2099 (11.2%)	
2	4471 (26.5%)	4697 (25.1%)	
3	2416 (14.3%)	2320 (12.4%)	
4	872 (5.2%)	915 (4.9%)	
Ejection fraction, *n* (%)			<0.0001
Normal >60%	10,852 (63.7%)	12,624 (67.5%)	
Mild 46%–60%	4049 (23.8%)	4006 (21.4%)	
Moderate 30%–45%	1667 (9.8%)	1593 (8.5%)	
Severe <30%	481 (2.8%)	489 (2.6%)	

Abbreviations: BMI, body mass index; CSS, Canadian Cardiovascular Society; eGFR, estimated glomerular filtration rate; SD, standard deviation.

**FIGURE 1:**
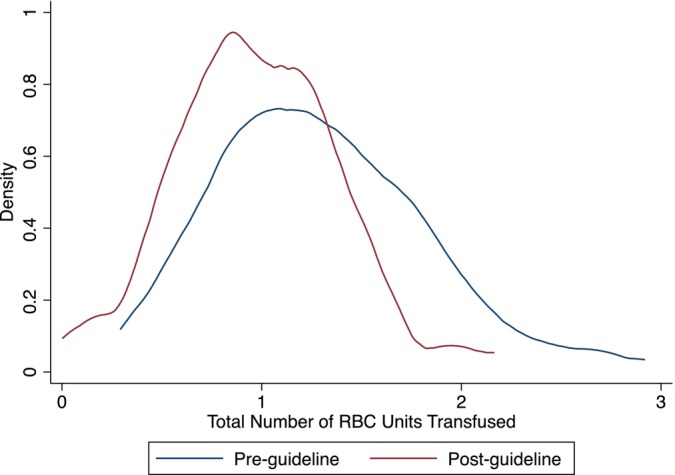
Distribution of the total red blood cell (RBC) units transfused across 80 surgeons.

**FIGURE 2:**
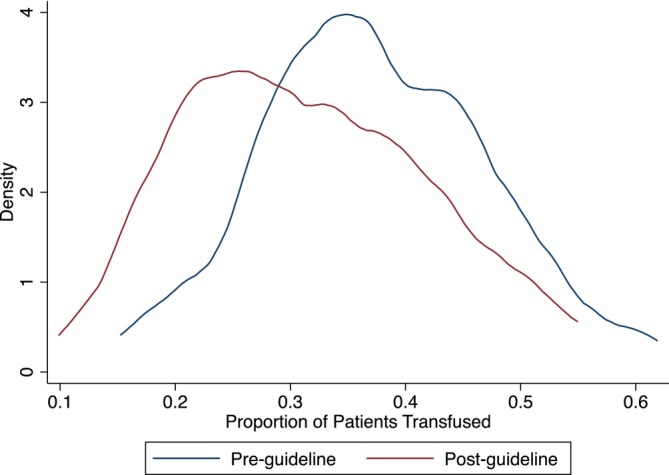
Distribution of the proportion of patients transfused across 80 surgeons.

**FIGURE 3:**
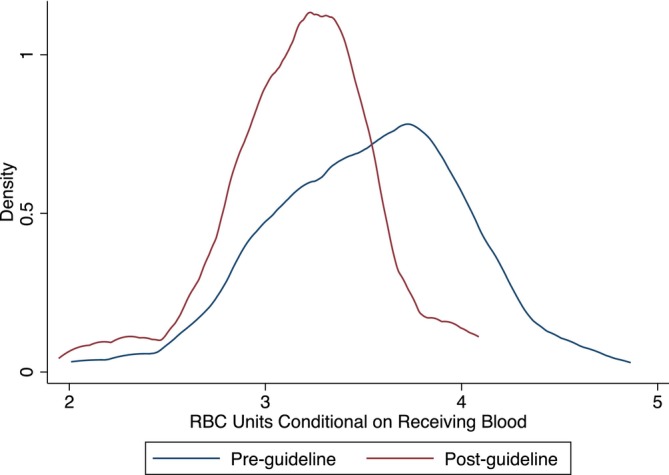
Distribution of red blood cell (RBC) units transfused across 80 surgeons, conditional on receiving blood.

Table 2 presents the results of the primary, elective surgery analysis. Full regression outputs are presented in the Appendix S1. Figures 1, 2, 3 present the corresponding results graphically as distributions of surgeon fixed effects.

**TABLE 2:** Primary analysis results—elective surgeries.

Outcome	Mean				Variation across surgeons^a^			
	Pre	Post	Change	*p*‐Value	Pre	Post	Change	*p*‐Value
Total RBC units transfused	1.29	0.98	−24.5%	<0.0001	0.271	0.170	−37.2%	0.040
Proportion of patients transfused	0.37	0.31	−15.9%	0.0003	0.009	0.011	17.7%	0.471
RBC units transfused conditional on receiving blood	3.53	3.18	−9.9%	<0.0001	0.253	0.155	−38.8%	0.030

Abbreviations: Pre, pre‐guideline; Post, post‐guideline; RBC, red blood cells.

^a^ After controlling for observed patient heterogeneity and sampling error.

Mean RBC use measured by all three metrics decreased significantly after the guidelines were published—15.9% fewer patients received RBCs (Figure 2) and those patients who did were transfused with 9.9% fewer units (Figure 3), leading to a 24.5% decrease in the total number of RBC units transfused (Figure 1). Reductions in mean RBC use are graphically represented by downward shifts in each of the distributions of surgeon fixed effects.

Variation in total RBC units across surgeons decreased significantly by 37.2% driven entirely by the number of RBC units transfused, conditional on receiving blood. There was no significant change in variation in the proportion of patients transfused. Reductions in variation are graphically represented as a narrowing of the distribution of surgeon fixed effects, observable in Figures 1 and 3, but not in Figure 2. This highlights that the reduction in variation in total RBC use is driven by surgeons becoming significantly more consistent in the number of RBCs units they transfused to those patients who received blood rather than in their propensity to use RBCs at all.

Table 3 presents the results of the secondary, non‐elective surgery analysis. Average RBC use was higher in non‐elective surgeries—a greater proportion of patients received transfusions and those who did received more units. After the guidelines were published, mean use and variation across surgeons in non‐elective patients exhibited similar changes to those observed in the primary analysis. There were significant reductions in mean use and variation across surgeons for total RBC units and RBC units, conditional on receiving blood. The mean proportion of patients transfused also decreased but variation across surgeons in this outcome remained unchanged. Compared with elective surgeries, decreases in variation were smaller in non‐elective surgeries.

**TABLE 3:** Secondary analysis results—non‐elective surgeries.

Outcome	Mean				Variation across surgeons^a^			
	Pre	Post	Change	*p*‐Value	Pre	Post	Change	*p*‐Value
Total RBC units transfused	3.40	3.01	−11.4%	<0.0001	0.314	0.248	−21.2%	0.307
Proportion of patients transfused	0.66	0.59	−9.3%	0.0001	0.007	0.010	40.9%	0.983
RBC units transfused conditional on receiving blood	4.78	4.28	−10.5%	<0.0001	0.370	0.241	−35.0%	0.066

Abbreviations: Pre, pre‐guideline; Post, post‐guideline; RBC, red blood cells.

^a^ After controlling for observed patient heterogeneity and sampling error.

## DISCUSSION

We quantified variation in RBC transfusions across surgeons before and after the publication of clinical guidelines promoting the appropriate use of blood. After the guidelines were published there was a 24.5% decrease in average RBC use accompanied by a 37.2% decrease in variation across surgeons. This change in variation was driven by the number of units transfused conditional on receiving blood rather than the proportion of patients transfused. The reduction in variation reflects that on average, surgeons with higher pre‐guideline RBC use exhibited larger reductions in the number of units transfused. Higher pre‐guideline RBC use is likely to be associated with more unnecessary transfusions and therefore more room for improvement from implementing the guideline's recommendations.

The results of the secondary analysis suggest after that publication of the guidelines there was a smaller improvement in the consistency of transfusion practices across surgeons when they were operating non‐electively, compared with electively. This aligned with a priori expectations, as many of the guideline's recommendations concern managing pre‐operative care and thus this result provides some evidence that the changes observed may be a causal effect of the guidelines.

We have previously published an interrupted time series analysis of the impact of the PBM guidelines on health outcomes using the same cardiac surgery registry [12]. This study found no difference in 30‐day hospital readmissions, 30‐day mortality or intensive care unit length of stay but did find a significant reduction in hospital length of stay after the guidelines were published. Without a suitable control group, the analysis is unable to draw a strong causal conclusion; however, it is reassuring to note that the reduction in variation in RBC transfusions observed in the current study was accompanied by an improvement in hospital length of stay. This finding supports the idea that there was unwarranted variation in care before the guidelines were published and that by providing evidence‐based recommendations on best practice, publishing guidelines can reduce unwarranted variation, improve the overall quality of care and minimize unnecessary resource use.

Our study is limited by several factors. Primarily, it is observational, and as such, we are unable to make strong conclusions about the causal effect of the guidelines. A significant body of PBM research was developed around the time the Australian guidelines were published such as the transfusion requirements after cardiac surgery clinical trial of restrictive transfusion strategies published in 2010 [18]. Our study design cannot rule out the influence of this and other contemporaneous confounding factors. Clinical guidelines such as those under evaluation in this study are one of the methods for translating positive findings from clinical trials into routine clinical practice. This type of dissemination plays a crucial role in reducing the time it takes for healthcare providers to integrate evidence‐based best practices. It may be that the effect observed in this study is a result of the broader push within the medical community towards PBM. However, the previous published interrupted time series analysis did identify a significant change in Australian practice specifically around the time these guidelines were published [12], and nonetheless, we believe the influence of PBM on variation in care in general is itself an interesting question.

To improve the accuracy of our estimates, we restricted our data to surgeons who performed at least 10 elective surgeries in the pre‐ and post‐guideline periods. As a result, we excluded a proportion of surgeons who may have been new to the database, potentially younger and more likely to adhere to new evidence. Others will have operated in low‐frequency hospitals where standards may not align with best practice. The size and direction of any bias from the data restrictions is unknown. Given the limitations of our data, we attribute any effect of the guidelines on transfusion decisions to the operating surgeon; however, these decisions are regularly made by the operating team, including the anaesthetist and intensivist. Moreover, without any characteristics of the cardiac surgeons we cannot explain the heterogeneous responses to the guidelines across surgeons. Surgeon age, gender and experience may influence adherence to guidelines. Finally, there are limitations of the ANZSCTS database that precluded additional analyses such as determining the specific PBM recommendations that changed practice. The data does not include any information about the indication for transfusion and the only recommendation for which we had data was for tranexamic acid, which was not well recorded and resulted in highly uncertain surgeon effects. The ANZSCTS Database does record intra‐operative and post‐operative haemoglobin which could be used to assess the impact of transfusion triggers; however, these parameters are recent additions to the Database and were not available during our data window.

The main strength of our study is the large national dataset and the novel method with which we were able to quantify variation. We estimated variation in a setting where there may be correlation between the unit‐level effects and patient risk factors, violating the random effects assumption. We recommend that where possible all clinical guidelines undergo a retrospective quantitative assessment of their impact on both average use and variation in care, as these two measures are statistically independent. If effective, average use will move in the intended direction after implementation of clinical guidelines. In our example, the PBM clinical guidelines were intended to reduce RBC transfusions; however, other clinical guidelines could intend to increase the use of an underutilized resource. Nonetheless, the intended effect on variation in care is always a decrease. Given the statistical independence, there may well be settings in which clinical guidelines improve the average quality of care but also increase variation in care, highlighting the need to assess changes in both measures.

Providing consistent, appropriate care across the population is a core objective of health policy around the world [9]. Nevertheless, there is only a very small literature that quantifies the impact of an intervention designed to reduce variation in care. A 1999 US paper found that a national consensus conference that recommended breast conservation therapy for women with Stage I or II breast cancer had no effect on regional variation in care [19]. More recently, a 2011 Dutch paper studying regional and temporal variations in breast cancer treatments found increased compliance with national guidelines resulting in a reduction in regional variation [20]. To the best of our knowledge, most published studies looking at the effects of interventions designed to reduce variation in transfusion practices only quantify the change in average use, and not the change in variation. Furthermore, none of the published studies identified attempt to control for sampling error, a concept which is perhaps not as well understood among the clinical community as it is among statisticians [21].

This study shows that it is feasible to assess the change in both average use and variation in care of interventions designed to improve the quality of care. Notwithstanding the causal limitations of our analysis, the results suggest that clinical guidelines can be effective in improving the consistency of perceived best practice care in transfusion medicine. When designing and implementing such interventions, policymakers are advised to consider whether appropriate data are being collected to facilitate quantitative assessments of their impact. Further research is warranted to determine the most cost‐effective method for reducing variation such as clinical guidelines, incentives or regulation.

In conclusion, we quantitatively demonstrate that in the setting of cardiac surgery in Australia, variation in transfusion practices across surgeons decreased after the publication of national clinical guidelines designed to reduce inappropriate blood transfusions. We hypothesise that this pattern of response occurred because the guidelines elicited larger reductions in transfusions from surgeons with relatively high pre‐guideline transfusion rates.

## CONFLICT OF INTEREST STATEMENT

The authors declare no conflicts of interest.

## Supporting information


**Data S1.** Supporting Information.

## Data Availability

The data used in this project are publicly available via an application to the Australian & New Zealand Society of Cardiac & Thoracic Surgeons steering committee.
