# Supplementary material for: Can clinical guidelines reduce variation in transfusion practice? A pre–post study of blood transfusions during cardiac surgery
Source: Vox Sang. 2024 Oct 14;120(1):47–54. doi: 10.1111/vox.13751 (PMC11753820; doi:10.1111/vox.13751)
Supplement: Supplementary file 1 — Data S1. Supporting Information. [file VOX-120-47-s001.docx]

##### Supplementary Appendix

Statistical Analysis

Variation across surgeons was estimated in two steps. In the first step, we estimated a linear regression model including observed patient risk factors and surgeon fixed effects.

$y_{ijt}= \alpha+ {\beta X}_{i}+{u_{j0}+u_{j1}+ \delta}_{ij}$ $\delta_{ij} \sim N(0, \sigma_{\varepsilon}^{2})$

Where$y_{ij}$ is an indicator representing the RBC use of patient $i$ prescribed by surgeon$j$ in period $t$ ($t$ takes 0 and 1 in the pre- and post-guideline periods, respectively). The intercept is represented by $\alpha$ and ${\beta X}_{i}$ is the vector of observed patient risk factors identified from a previously published predictive model of RBC transfusions.[5] The regression included separate surgeon fixed effects for the pre- and post-guideline periods, $u_{j0}$ and $u_{j1}$ respectively. A complete list of the included observed patient risk factors and the regression results are presented in the Supplementary Appendix.

In the second step, we performed an empirical Bayes shrinkage of the surgeon fixed effects. We assumed there is one true surgeon effect in each periodand that the observed surgeon effects are the true surgeon effect plus the uncorrelated sampling error. Each observed fixed effect was then re-estimated with a prior beta distribution informed by the empirical data, ‘shrinking’ them towards the overall mean across all surgeons, reducing the sampling error and its variance. The shrinkage was done separately and identically for both the pre- and post-guideline surgeon fixed effects using the estimator proposed by Morris[16] using a publicly available Stata program.[17] The statistical significance of the change in variation was assessed using the variance ratio F-test.

Kernel density plots are scaled such that the area under the curve is equal to unity and as such the y-axes of the plots are not meaningfully interpretable. These graphs are useful for visually comparing the shapes of distributions, as is required in this study. Kernel density plots can be used to represent the distribution of the use of any resource across any units of the healthcare system. In our figures, the resource use is RBC transfusions, measured by our three metrics, and the unit of the healthcare system is cardiac surgeon. The width of the kernel density plot represents variation across surgeons, the peak of plot represents the mode, and the centre of the plot (50^th^ percentile) represents the median. As the area under a kernel density plot must always equal unity, if variation (the width of plot) decreases, then by design average use (the peak of the plot) must increase. This would represent more consistent resource use and a reduction in variation in care. If, however, the width remains the same, and the curve shifts uniformly downwards, average resource use has decreased but variation in care has remained the same. These two examples are illustrative of potential outcomes and highlight the statistical independence of changes in average use from changes in variation.

Table A.3: Primary Analysis Regression Results

|  | Total number of RBC units transfused | Proportion of patients who received an RBC transfusion | Number of units transfused, conditional on receiving RBCs | |
| --- | --- | --- | --- | --- |
| Age | -0.003  (0.002) | -0.001***  (0.000) | -0.013***  (0.004) |  |
| Male | -0.404***  (0.039) | -0.177***  (0.006) | -0.383***  (0.080) |  |
| BMI | -0.010***  (0.003) | -0.005***  (0.001) | -0.009  (0.007) |  |
| Cardiac catheterisation | -0.547***  (0.094) | -0.056***  (0.010) | -1.024***  (0.234) |  |
| Cardiogenic shock | 2.433***  (0.610) | 0.240***  (0.035) | 1.943**  (0.776) |  |
| Congestive heart failure | 0.386  (0.050) | 0.043***  (0.007) | 0.477***  (0.099) |  |
| Cerebrovascular disease | 0.211***  (0.067) | 0.034***  (0.008) | 0.196  (0.130) |  |
| Diabetes | 0.124***  (0.038) | 0.046***  (0.006) | -0.067***  (0.086) |  |
| ln(eGFR) | -1.084***  (0.063) | -0.215***  (0.008) | -0.774***  (0.115) |  |
| Infective endocarditis | 1.620***  (0.217) | 0.215***  (0.019) | 1.272***  (0.352) |  |
| Myocardial infarction | 0.068  (0.044) | 0.028***  (0.006) | 0.008  (0.096) |  |
| Peripheral vascular disease | 0.380***  (0.081) | 0.053***  (0.009) | 0.429***  (0.149) |  |
| Respiratory disease | 0.197***  (0.056) | 0.025***  (0.007) | 0.243**  (0.116) |  |
| Dialysis | 0.312  (0.245) | 0.011  (0.025) | 0.097  (0.326) |  |
| Intra-aortic balloon pump | 3.167***  (0.295) | 0.216***  (0.016) | 3.387***  (0.398) |  |
| Previous cardiac surgery | 0.468***  (0.052) | 0.056***  (0.006) | 0.601***  (0.111) |  |
| Angina – CCS classification |  |  |  |  |
| 1 | -0.129**  (0.051) | -0.007  (0.008) | -0.351***  (0.121) |  |
| 2 | -0.035  (0.046) | 0.011  (0.007) | -0.195*  (0.106) |  |
| 3 | 0.017  (0.060) | 0.022***  (0.008) | -0.139  (0.137) |  |
| 4 | 0.102  (0.085) | 0.033***  (0.011) | -0.051  (0.175) |  |
| Ejection fraction |  |  |  |  |
| Mild 46-60% | 0.019  (0.040) | 0.003  (0.006) | 0.032  (0.096) |  |
| Moderate 30-45% | -0.028  (0.065) | 0.022**  (0.009) | -0.231*  (0.136) |  |
| Severe <30% | -0.086  (0.170) | 0.034***  (0.016) | -0.332  (0.332) |  |
| Coronary artery bypass | 0.419***  (0.067) | 0.120***  (0.008) | -0.030  (0.133) |  |
| Valve surgery | 0.577***  (0.064) | 0.120***  (0.008) | 0.444***  (0.127) |  |
| Fixed effects | Surgeon^§^ | Surgeon^§^ | Surgeon^§^ |  |

***p<0.01, **p<0.05, *p<0.10,

^§^ Regression includes two sets of surgeon fixed effects – one for the pre-guideline period and one for the post-guideline period

BMI = body mass index; CCS = Canadian Cardiovascular Society; eGFR = estimated Glomerular filtration rate

Table A.3: Patient Blood Management Clinical Guideline Recommendations

| **No.** | **Recommendation** |
| --- | --- |
| R1 | Health-care services should establish a multidisciplinary, multimodal perioperative patient blood management program (Grade C). This should include preoperative optimisation of red cell mass and coagulation status; minimisation of perioperative blood loss, including meticulous attention to surgical haemostasis; and tolerance of postoperative anaemia. |
| R2 | In patients undergoing cardiac surgery, preoperative anaemia should be identified, evaluated and managed to minimise RBC transfusion, which may be associated with an increased risk of morbidity, mortality, ICU length of stay and hospital length of stay (Grade C). |
| R3 | In patients undergoing noncardiac surgery, preoperative anaemia should be identified, evaluated and managed to minimise RBC transfusion, which may be associated with an increased risk of morbidity, mortality, ICU length of stay and hospital length of stay (Grade C). |
| R4 | In surgical patients with, or at risk of, iron deficiency anaemia, preoperative oral iron therapy is recommended (Grade B). |
| R5 | In patients with preoperative anaemia, where an ESA is indicated, it must be combined with iron therapy (Grade A). |
| R6 | In patients with postoperative anaemia, early oral iron therapy is not clinically effective; its routine use in this setting is not recommended (Grade B). |
| R7 | In patients undergoing CABG either with or without CPB (OPCAB), clopidogrel therapy should be stopped, where possible, at least 5 days before surgery (Grade C). |
| R8 | In patients undergoing noncardiac surgery, it is reasonable to continue low dose aspirin therapy. This may require specific evaluation in neurosurgery and intraocular surgery (Grade C). |
| R9 | In patients undergoing elective orthopaedic surgery, NSAID therapy should be ceased preoperatively to reduce blood loss and transfusion (Grade C). The timing of the cessation should reflect the agent’s pharmacology. |
| R10 | In patients undergoing minor dental procedures, arthrocentesis, cataract surgery, upper gastrointestinal endoscopy without biopsy or colonoscopy without biopsy, warfarin may be continued (Grade B). |
| R11 | The *routine* use of preoperative autologous donation is not recommended because, although it reduces the risk of allogeneic RBC transfusion, it increases the risk of receiving any RBC transfusion (allogeneic and autologous) (Grade C). |
| R12 | In patients undergoing surgery, measures to prevent hypothermia should be used (Grade A). |
| R13 | In patients undergoing radical prostatectomy or major joint replacement, if substantial blood loss (blood loss of a volume great  enough to induce anaemia that would require therapy) is anticipated, deliberate induced hypotension (MAP 50–60 mmHg) should be considered, balancing the risk of blood loss and the preservation of vital organ perfusion (Grade C). |
| R14 | In adult patients undergoing surgery in which substantial blood loss (blood loss of a volume great enough to induce anaemia that would require therapy) is anticipated, the use of ANH should be considered (Grade C). |
| R15 | In adult patients undergoing surgery in which substantial blood loss (blood loss of a volume great enough to induce anaemia that would require therapy) is anticipated, intraoperative cell salvage is recommended (Grade C). |
| R16 | In adult patients undergoing cardiac surgery, the use of TEG should be considered (Grade C). |
| R17 | In adult patients undergoing cardiac surgery, the use of intravenous tranexamic acid is recommended (Grade A). |
| R18 | In adult patients undergoing noncardiac surgery, if substantial blood loss (blood loss of a volume great enough to induce anaemia that would require therapy) is anticipated, the use of intravenous tranexamic acid is recommended (Grade B). |
| R19 | In adult patients undergoing cardiac surgery, the use of intravenous ε-aminocaproic acid is recommended (Grade C). |
| R20 | In adult patients undergoing cardiac surgery or total knee arthroplasty, in whom significant postoperative blood loss is anticipated, postoperative cell salvage should be considered (Grade C). |
| R21 | The prophylactic use of FFP in cardiac surgery is not recommended (Grade B). |
| R22 | The prophylactic or routine therapeutic use of rFVIIa is not recommended because concerns remain about its safety profile, particularly in relation to thrombotic adverse events (Grade C). |

ANH, acute normovolemic haemodilution; CABG, coronary artery bypass surgery; CPB, cardiopulmonary bypass; ESA, erythropoiesis-stimulating agent; FFP, fresh frozen plasma; ICU, intensive care unit; NSAID, nonsteroidal anti- inflammatory drug; MAP, mean arterial blood pressure; OPCAB, off-pump coronary artery bypass; RBC, red blood cell; rFVIIa, recombinant activated factor VIIa; TEG, thromboelastography
